# Tissue-Specific miRNA Responses to Tick-Borne Encephalitis Virus Strains in Mice

**DOI:** 10.3390/ncrna12050042

**Published:** 2026-10-20

**Authors:** Elena V. Mikheeva, Galina S. Chicherina, Kseniya S. Aulova, Georgy A. Nevinsky, Anna M. Timofeeva

**Affiliations:** 1 SB RAS Knorre Institute of Chemical Biology and Fundamental Medicine, 630090 Novosibirsk, Russiak.aulova@alumni.nsu.ru (K.S.A.);; 2 Institute of Systematics and Ecology of Animals of the Siberian Division of the RAS, 630091 Novosibirsk, Russia

**Keywords:** tick-borne encephalitis virus, microRNA, miRNA, tissue specificity, neuroinvasion, strain specificity, vaccination, biomarkers, NF-κB signaling, stem-loop RT-qPCR, Tick-E-Vak, Aina strain, Sofyin strain, innate immunity, proinflammatory cascade, plasma biomarkers, neurovirulence, immune response, circulating miRNA

## Abstract

**Background/Objectives**: Tick-borne encephalitis virus (TBEV) is a vector-borne flavivirus that targets the CNS and causes severe encephalitis with long-term neurological sequelae. As the geographic range of its vectors expands and climate conditions shift, the incidence of tick-borne encephalitis continues to rise, yet no specific antiviral therapy has been developed to date. Understanding the molecular mechanisms of tissue-specific immune responses during TBEV infection is essential for identifying novel therapeutic targets and biomarkers. **Methods**: In this study, quantitative reverse transcription PCR with stem-loop primers was used to examine changes in the levels of seven immunoregulatory miRNAs (let-7i, miR-9-5p, miR-21a-5p, miR-30a-5p, miR-146b-5p, miR-181a-5p, miR-486-5p) in blood plasma, peripheral organs (spleen, liver), and four central nervous system structures (olfactory bulb, cerebral hemispheres, cerebellum, spinal cord) of BALB/c mice. Three experimental groups were compared: (1) infection with the Aina strain (Siberian subtype), (2) infection with the Sofyin strain (Far Eastern subtype), and (3) vaccination with the vaccine “Tick-E-Vak”. **Results**: It was shown that both infection and vaccination induce pronounced tissue-specific changes in the miRNA profile. MiR-181a-5p is a negative regulator of TLR4/NF-κB signaling. Its downregulation was the most reproducible change observed across all compartments and in all three groups, suggesting a possible universal mechanism for derepression of the proinflammatory cascade. Two miRNAs (miR-30a-5p and miR-146b-5p) were altered in all examined tissues and may be considered candidate markers of antigenic stimulation. **Conclusions**: The direction of their changes in plasma and tissues was frequently opposite, suggesting redistribution of miRNAs between the tissue and circulating pools, which is important for the interpretation of plasma biomarkers. Infection-specific changes were identified for let-7i and miR-9-5p in the CNS. The most pronounced changes were observed in the structures most affected by neuroinvasion (olfactory bulb, spinal cord), making them candidate markers associated with TBEV infection and CNS involvement. Infection with the Aina strain induced more pronounced miRNA changes in peripheral organs and the CNS than the Sofyin strain, despite the higher epidemiological lethality of the latter. This suggests that the pathogenicity of individual strains in the mouse model may not always correspond to the subtype-based lethality classification in humans and is determined by strain-specific features of tropism and infection kinetics.

## 1. Introduction

Tick-borne encephalitis virus (TBEV) is a vector-borne flavivirus that targets the CNS and causes severe encephalitis with long-term neurological sequelae [1,2]. The major endemic foci of TBEV are located in the Russian Federation, where thousands of cases are registered annually. Today, mass vaccination remains the only strategy for reducing the disease incidence [3,4]. However, population immunity remains low even in highly endemic regions [5]. Although effective vaccines are available, none of them provides 100% protection, and specific antiviral drugs for the treatment of TBEV infection have not been developed to date [6]. The lethality of severe forms reaches 2–30% depending on the virus subtype, and a substantial proportion of survivors develop persistent neurological deficits [6,7], including those potentially associated with molecular mimicry [8]. These factors underscore the need for a deeper understanding of the molecular basis of TBEV pathogenesis, in particular, the differences between the Siberian and Far Eastern subtypes that predominate in Russia.

MicroRNAs (miRNAs) are small non-coding RNAs that regulate gene expression at the post-transcriptional level [9]. During viral infections, miRNAs are also involved in the antiviral response [10,11]. In vivo studies of endogenous miRNAs in flavivirus infections have focused primarily on Japanese encephalitis, West Nile, Zika, and dengue viruses [12,13]. A recent in vivo study demonstrated progressive miRNA dysregulation in the brains of mice infected with Powassan virus, a tick-borne flavivirus phylogenetically closely related to TBEV, indicating that the miRNA response is a significant component of pathogenesis in tick-borne flavivirus infections [14]. However, comparable studies for TBEV are virtually absent.

In recent years, accumulating evidence has shown that different strains of the same virus can elicit qualitatively distinct immune responses. For Zika virus, African and Asian strains induce different levels of pro-inflammatory cytokines in the mouse brain and differ in disease severity, while viral load does not correlate with pathogenicity [15]. For influenza virus, H1N1 and H5N1 strains differ in the kinetics of interferon production and the magnitude of immune infiltration, which are the main drivers of strain-specific immunodynamics and infection outcomes [16], and closely related isolates of pandemic H1N1 differ in their cytokine profiles, which correlate with virulence [17]. SARS-CoV-2 variants (Alpha, Beta, Delta, Omicron) elicit both shared and variant-specific transcriptional responses in the lungs [18]. These data indicate that strain-specific immune response is a universal phenomenon that determines the outcomes of viral infections. However, the miRNA response underlying these differences remains largely uncharacterized for most viruses, and TBEV is no exception.

Until recently, TBEV was considered a single species divided into five subtypes (European, Siberian, Far Eastern, Baikal, and Himalayan) that differ substantially in virulence and clinical outcomes [6,7]. In February 2026, ICTV ratified a taxonomic reclassification, under which the European subtype was assigned to a separate species, *Orthoflavivirus neudoerflense*, while the remaining subtypes were assigned to the species *Orthoflavivirus zilberi* [19]. Despite the clinical significance of subtype differences, the molecular mechanisms of strain-specific TBEV pathogenesis, in particular the changes in miRNA profiles upon infection with different strains, remain insufficiently characterized.

miRNA concentrations differ substantially between tissues: out of ~1300 detected miRNAs in mice, approximately 400 are differentially expressed across 11 tissues, with the brain containing the largest number of unique transcripts [20]. In neurotropic flavivirus infections, the miRNA response in the brain differs from that in the periphery. For example, Zika virus infection in neurons causes pronounced suppression of brain-enriched miRNAs [21]. Thus, the miRNA response to flavivirus infection is shaped in a tissue-specific manner. Since TBEV is a neurotropic virus, comparing miRNA profiles across peripheral organs, CNS structures, and plasma is necessary for a comprehensive understanding of pathogenesis and for identifying compartment-specific targets.

A separate question is the comparison of the miRNA response to natural infection and vaccination. It is well established that immunity induced by inactivated vaccines differs qualitatively from that elicited by natural infection [22]. During infection, both Th1 and Th2 CD4+ T cells and CD8+ T cells are activated, whereas inactivated vaccines induce a predominantly Th2 response [23]. Since miRNA signatures can distinguish protective from pathogenic immune reactions [24], and differential miRNA profiles between vaccination and infection have been demonstrated for other pathogens [25,26], comparing the miRNA response to live infection and vaccination in the context of TBEV is of considerable scientific interest.

The aim of this study is to determine how infection with two TBEV strains (Sofyin, Far Eastern subtype; Aina, Siberian subtype) and vaccination with the “Tick-E-Vak” vaccine affect miRNA concentrations in plasma, peripheral organs, and CNS structures of mice, and to identify tissue-specific, strain-specific, and vaccine-specific miRNA signatures of the immune response.

## 2. Results

Tissue-specific changes in the levels of seven miRNAs (miR-9-5p, miR-30a-5p, miR-181a-5p, miR-146b-5p, miR-486-5p, miR-21a-5p, and let-7i) were analyzed upon infection with two TBEV strains in BALB/c mice. The seven miRNAs included in the panel were selected based on three criteria. First, all of them are established immunoregulatory miRNAs with documented involvement in the control of innate immune and antiviral responses: miR-146b-5p and miR-181a-5p are negative regulators of the TLR/NF-κB cascade [10,11,27], let-7i directly targets TLR4 mRNA [28,29], miR-30a-5p controls autophagy via Beclin-1 [30,31], and miR-21a-5p is involved in inflammatory resolution [32,33]. Second, the panel overlaps with the miRNAs previously characterized by our group in the plasma of patients with different clinical forms of TBE [34], enabling direct comparison between the mouse model and human data. Third, for all seven miRNAs, the mature sequences and seed regions are conserved between mouse and human, supporting the translational relevance of the findings. Two TBEV strains representing epidemiologically significant subtypes were analyzed: Sofyin and Aina. The Sofyin strain belongs to the Far Eastern subtype. The “Tick-E-Vak” vaccine is a formalin-inactivated cell culture vaccine prepared from the Sofyin strain. The Aina strain belongs to the Siberian subtype, which predominates in most endemic territories of Russia. Profiling was performed across blood plasma, peripheral organs, and distinct CNS structures. To characterize the miRNA response elicited by protective immunization, the analysis included a group of mice vaccinated with the “Tick-E-Vak” vaccine. The control group consisted of mice immunized with PBS.

### 2.1. Blood Plasma and Peripheral Organs

Blood plasma, spleen, and liver were selected to capture miRNA changes at different levels of the immune response. Blood plasma reflects the systemic circulating pool of miRNAs and is considered a potential source of non-invasive biomarkers of infection. The spleen and liver were selected as the organs where innate and adaptive immune responses are shaped: the spleen is the largest secondary lymphoid organ and the central site of antigen-dependent activation of B and T lymphocytes, while the liver plays a central role in the systemic inflammatory response [35].

**Figure 1:**
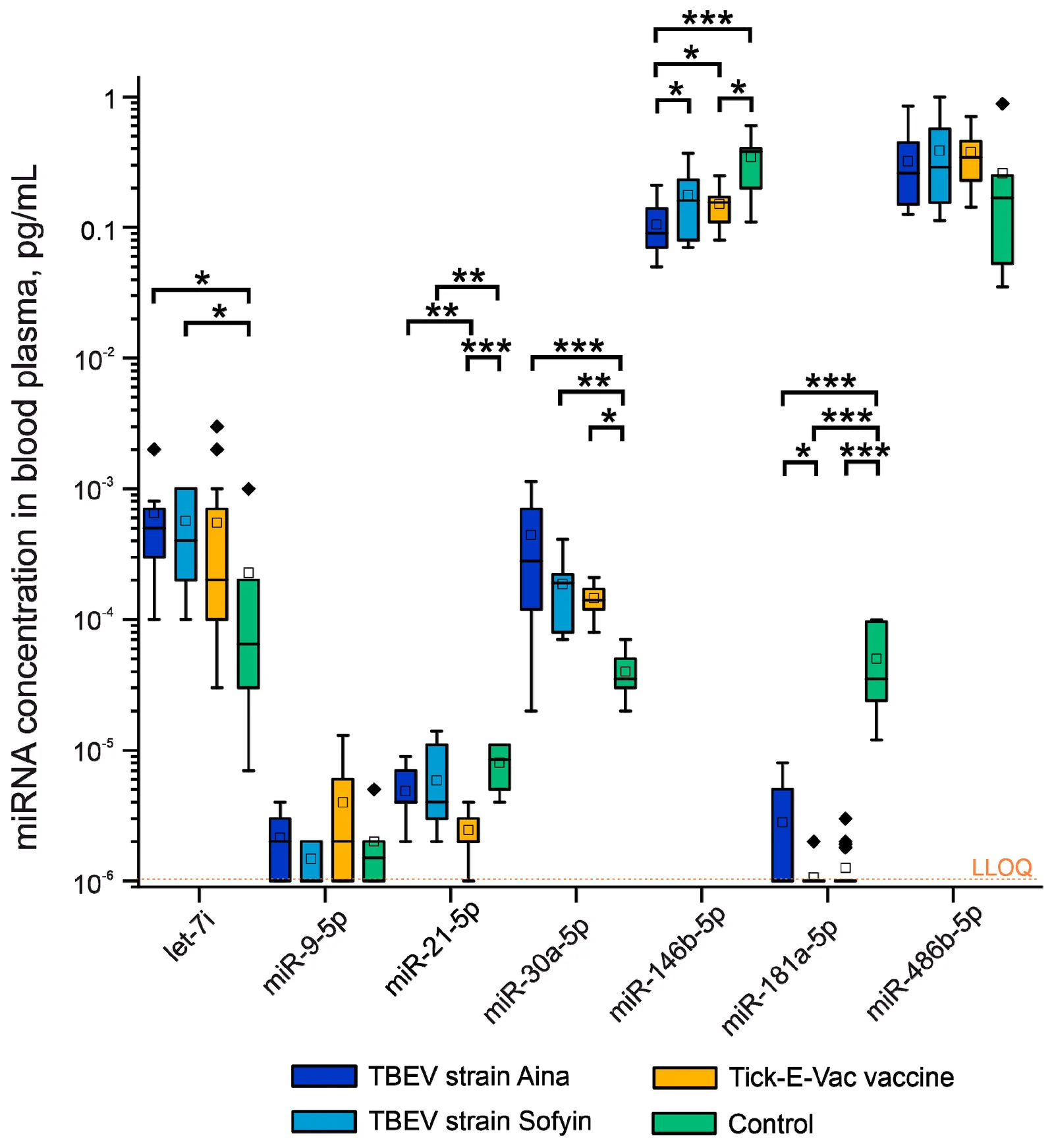
Analysis of miRNA concentrations in blood plasma of mice infected with the Aina and Sofyin strains, and vaccinated with “Tick-E-Vak”. Differences between groups were assessed using the Kruskal–Wallis test; pairwise comparisons were performed using Dunn’s test. The dashed line indicates the lower limit of quantification (LLOQ). Significance levels: * *p* < 0.05, ** *p* < 0.01, *** *p* < 0.001.

Upon viral infection, significant changes in plasma miRNA concentrations relative to the control were detected for four miRNAs, three of which (let-7i, miR-30a-5p, and miR-181a-5p) were altered in both infected groups. Following vaccination with the “Tick-E-Vak” vaccine, significant differences from the control were also detected for four miRNAs: miR-21a-5p, miR-30a-5p, miR-146b-5p, and miR-181a-5p (Figure 1).

In blood plasma, miR-30a-5p concentration was elevated in all three experimental groups, with the most pronounced increase upon infection with the Aina strain (8.0-fold, *p* = 0.0007) and the least pronounced upon vaccination (4.0-fold, *p* = 0.017). Infection with the Sofyin strain produced a 5.5-fold increase *(p* = 0.005). The magnitude of the increase declined in the following order: Aina infection > Sofyin infection > vaccination. MiR-181a-5p concentration, in contrast, was significantly decreased in all three groups, suggesting a common regulatory mechanism for this miRNA during both natural infection and vaccination.

miR-146b-5p concentration decreased significantly upon infection with the Aina strain (4.2-fold, *p* = 0.001) and upon vaccination (2.4-fold, *p* = 0.041), but not upon infection with the Sofyin strain, where only a unidirectional trend was observed.

The increase in let-7i concentration was specific to viral infection: it was significantly elevated upon infection with the Aina strain (7.9-fold, *p* = 0.040) and the Sofyin strain (6.3-fold, *p* = 0.040), whereas vaccinated animals showed a similar trend that did not reach statistical significance. Conversely, the decrease in miR-21a-5p concentration was exclusive to vaccination: it was significantly reduced in vaccinated mice (4.2-fold, *p* = 0.0005), while both infected groups showed only a non-significant trend toward decrease.

**Figure 2:**
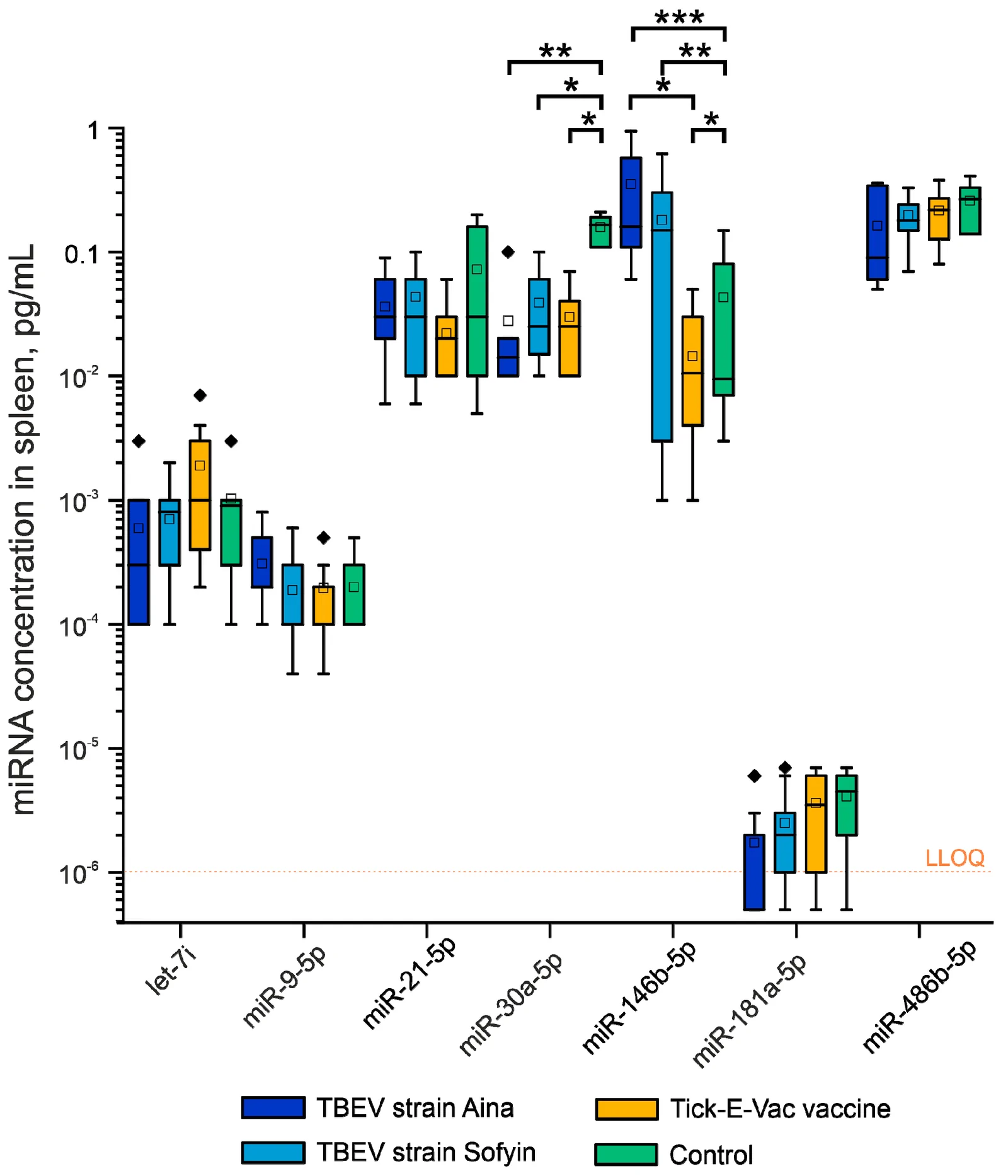
Analysis of miRNA concentrations in the spleen of mice infected with the Aina and Sofyin strains, and vaccinated with “Tick-E-Vak”. Differences between groups were assessed using the Kruskal–Wallis test; pairwise comparisons were performed using Dunn’s test. The dashed line indicates the lower limit of quantification (LLOQ). Significance levels: * *p* < 0.05, ** *p* < 0.01, *** *p* < 0.001.

In the spleen, only two miRNAs showed statistically significant changes. MiR-30a-5p was decreased in all three groups, with the most pronounced reduction upon infection with the Aina strain. MiR-146b-5p, in contrast, was increased in all three groups. However, the magnitude of this increase was substantially greater upon infection with both strains than upon vaccination (Figure 2).

**Figure 3:**
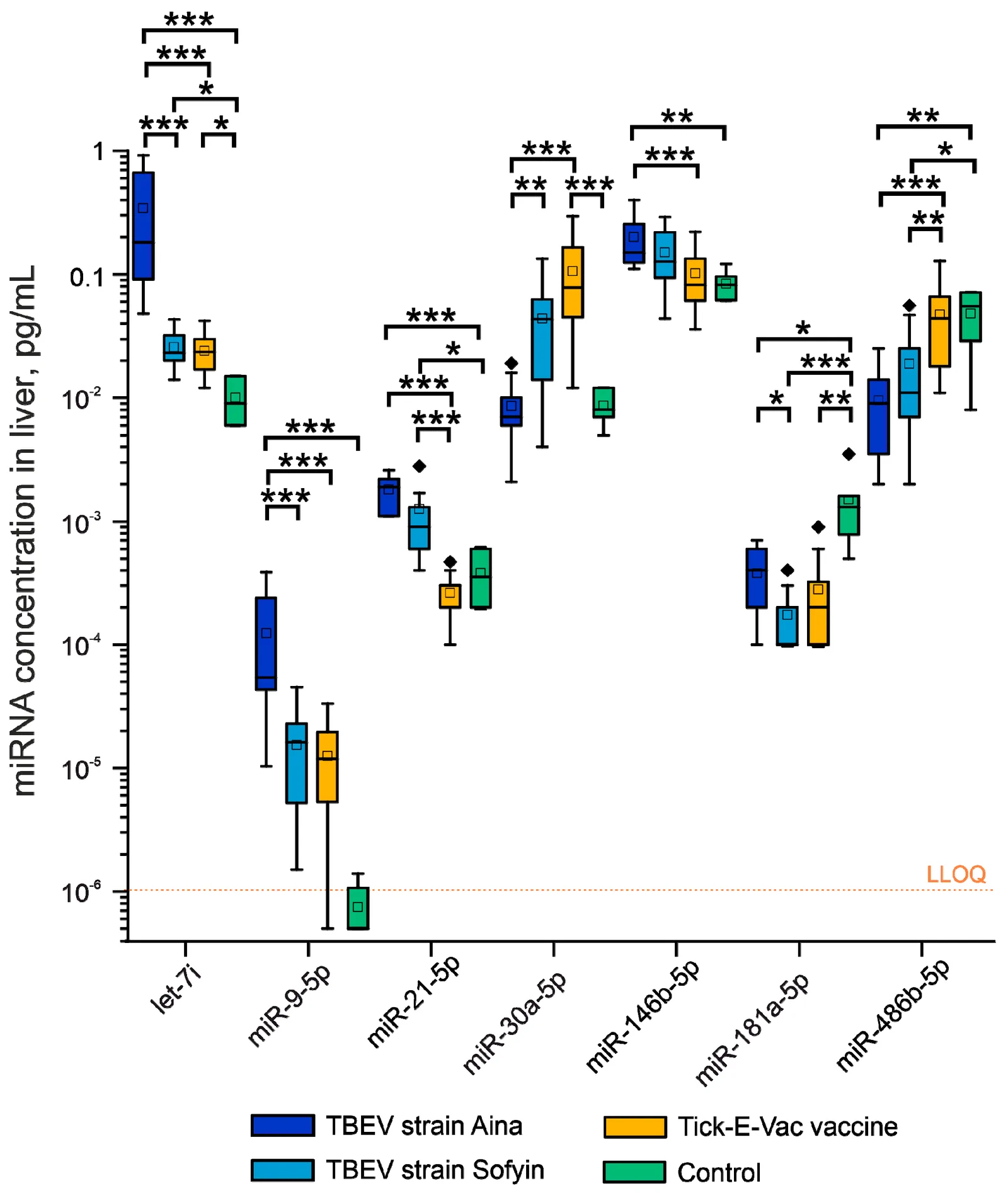
Analysis of miRNA concentrations in liver cord of mice infected with the Aina and Sofyin strains, and vaccinated with “Tick-E-Vak” Differences between groups were assessed using the Kruskal–Wallis test; pairwise comparisons were performed using Dunn’s test. The dashed line indicates the lower limit of quantification (LLOQ). Significance levels: * *p* < 0.05, ** *p* < 0.01, *** *p* < 0.001.

In the liver, let-7i, miR-21a-5p, miR-181a-5p, and miR-486-5p were significantly altered upon infection with both strains: let-7i (Aina: *p* < 0.0001; Sofyin: *p* = 0.026), miR-21a-5p (Aina: *p* = 0.0006; Sofyin: *p* = 0.030), miR-181a-5p (Aina: *p* = 0.020; Sofyin: *p* < 0.0001), and miR-486-5p (Aina: *p* = 0.004; Sofyin: *p* = 0.031). MiR-9-5p and miR-146b-5p were significantly altered only upon infection with the Aina strain *(p* < 0.0001 and *p* = 0.006, respectively), whereas no significant differences from the control were detected upon infection with the Sofyin strain. Following vaccination with the “Tick-E-Vak” vaccine, significant differences from the control were detected for three miRNAs: let-7i (*p* = 0.029), miR-30a-5p (*p* = 0.0008), and miR-181a-5p (*p* = 0.002) (Figure 3).

Let-7i and miR-181a-5p were altered in both viral infection and vaccination, but the magnitude of these changes followed different patterns. let-7i concentration was significantly increased in all three groups, with the most pronounced elevation upon infection with the Aina strain (20-fold, *p* < 0.0001), followed by the Sofyin strain (2.6-fold, *p* = 0.026) and vaccination (2.6-fold, *p* = 0.029), which were comparable to each other. MiR-181a-5p concentration, in contrast, was significantly decreased in all three groups, but the most pronounced reduction was observed upon infection with the Sofyin strain (13-fold, *p* < 0.0001), followed by vaccination (6.5-fold, *p* = 0.002) and then the Aina strain (3.3-fold, *p* = 0.020). Thus, for let-7i, the gradient of change magnitude followed the order Aina > Sofyin = vaccination, whereas for miR-181a-5p it was different: Sofyin > vaccination > Aina.

The increase in miR-21a-5p and the decrease in miR-486-5p were specific to viral infection, observed upon infection with both the Aina strain (miR-21a-5p: 6.0-fold, *p* = 0.0006; miR-486-5p: 6.1-fold, *p* = 0.004) and the Sofyin strain (miR-21a-5p: 2.9-fold, *p* = 0.030; miR-486-5p: 5.0-fold, *p* = 0.031). Upon vaccination, no significant differences from the control were detected for either miRNA.

Two miRNAs (miR-9-5p and miR-146b-5p) were altered exclusively upon infection with the Aina strain (miR-9-5p: 108-fold, *p* < 0.0001; miR-146b-5p: 1.8-fold, *p* = 0.006); such a high fold change for miR-9-5p is due to the extremely low concentration of this miRNA in the liver of control animals, which is at the limit of detection (median 5 × 10^−10^). Upon infection with the Sofyin strain and upon vaccination, no significant differences from the control were detected for either miRNA. The only miRNA whose alteration was specific to vaccination was miR-30a-5p: its concentration was significantly increased upon vaccination (9.8-fold, *p* = 0.0008), while infection with the Sofyin strain showed only a non-significant trend toward increase.

### 2.2. Central Nervous System

miRNA concentrations were quantified in four CNS structures of mice: the olfactory bulbs, cerebral hemispheres, cerebellum, and spinal cord. These structures were selected on the basis of their distinct roles in TBEV neuroinvasion, ranging from the olfactory bulbs, one of the primary gateways of viral entry into the CNS, to the regions with established neurotropism and clinically significant damage [36,37,38,39,40].

**Figure 4:**
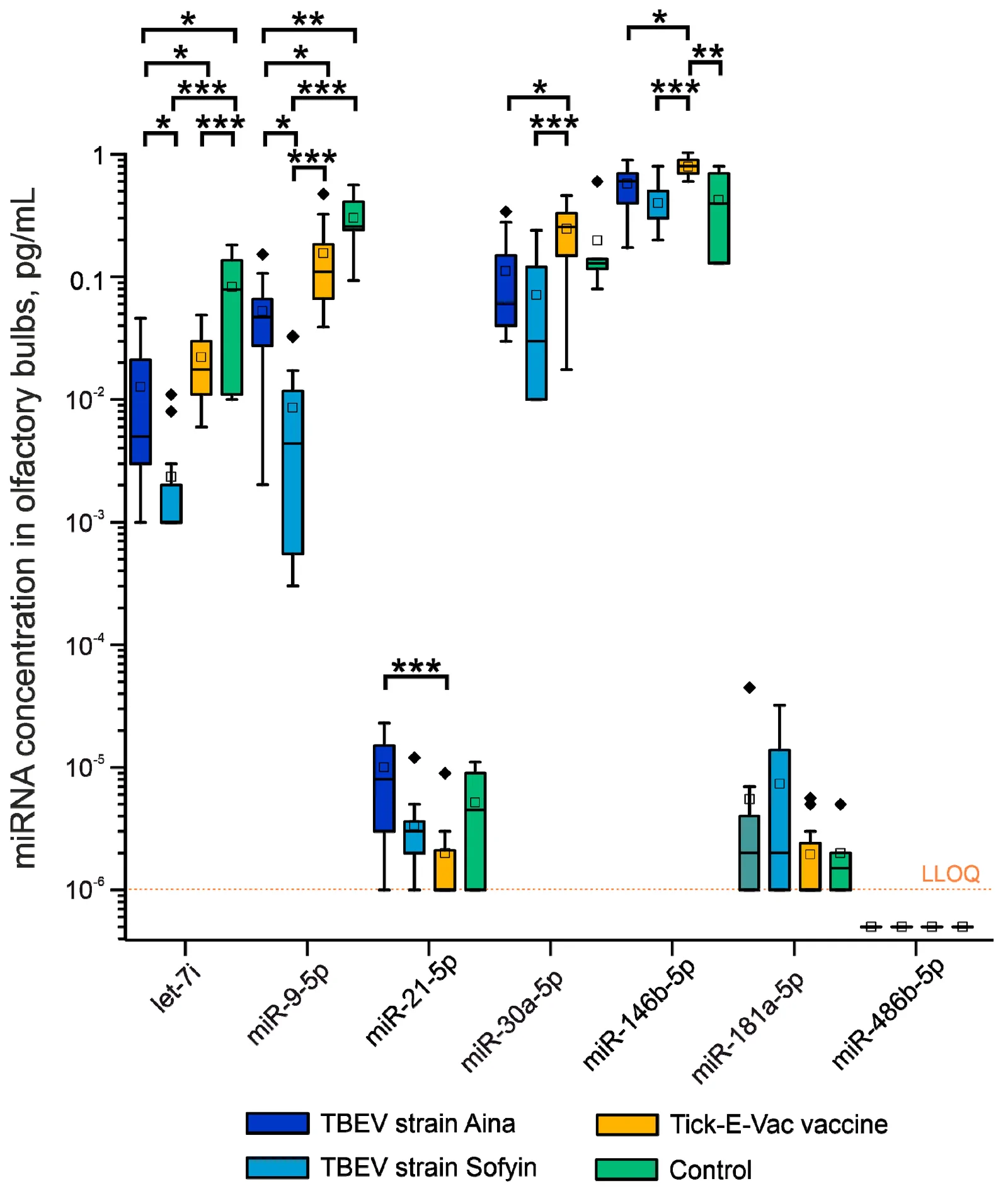
Analysis of miRNA concentrations in olfactory bulbs of mice infected with the Aina and Sofyin strains, and vaccinated with “Tick-E-Vak”. Differences between groups were assessed using the Kruskal–Wallis test; pairwise comparisons were performed using Dunn’s test. The dashed line indicates the lower limit of quantification (LLOQ). Significance levels: * *p* < 0.05, ** *p* < 0.01, *** *p* < 0.001.

In the olfactory bulbs, miR-486-5p concentration was below the detection limit in all animals. Upon viral infection, significant changes relative to the control were detected for let-7i and miR-9-5p in both infected groups (let-7i: Aina *p* = 0.032, Sofyin *p* < 0.0001; miR-9-5p: Aina *p* = 0.006, Sofyin *p* < 0.0001). Upon vaccination with the “Tick-E-Vak” vaccine, a significant difference from the control was detected only for miR-146b-5p *(p* = 0.007) (Figure 4).

Let-7i and miR-9-5p followed a common pattern: both miRNAs were significantly decreased upon infection with both strains, with the most pronounced reduction observed upon infection with the Sofyin strain. Let-7i and miR-9-5p followed a common pattern: both miRNAs were significantly decreased upon infection with both strains, with the most pronounced reduction observed upon infection with the Sofyin strain. let-7i concentration decreased 11.3-fold upon infection with the Aina strain (*p* = 0.032) and 56.5-fold upon infection with the Sofyin strain (*p* < 0.0001). MiR-9-5p concentration decreased 5.5-fold (*p* = 0.006) and 58.8-fold (*p* < 0.0001), respectively.

For miR-21a-5p and miR-30a-5p, no significant differences from the control were detected in any group; however, these two miRNAs showed significant differences between infected and vaccinated animals in opposite directions. MiR-21a-5p concentration was 8-fold higher upon infection with the Aina strain than upon vaccination (*p* < 0.0001), whereas miR-30a-5p concentration was 4.3-fold higher upon vaccination than upon infection with the Aina strain (*p* = 0.023) and 8.5-fold higher than upon infection with the Sofyin strain (*p* = 0.0002).

The only miRNA whose alteration was specific to vaccination was miR-146b-5p: its concentration was significantly increased upon vaccination (2.4-fold, *p* = 0.007), while no significant differences from the control were detected upon viral infection. The concentration in the vaccinated group was significantly higher than in both infected groups (*p* = 0.021 compared with the Aina group and *p* < 0.0001 compared with the Sofyin group).

**Figure 5:**
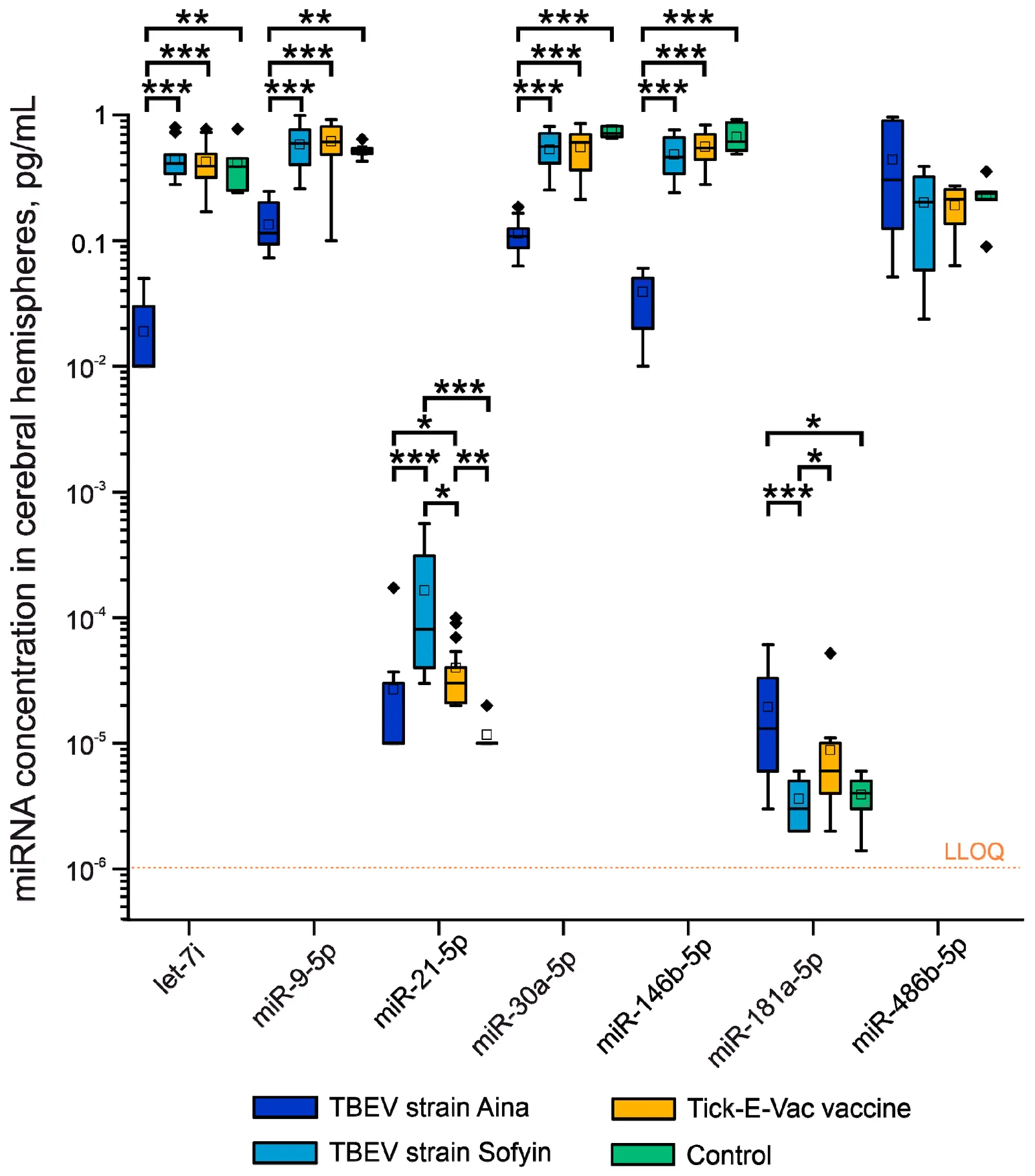
Analysis of miRNA concentrations in cerebral hemispheres of mice infected with the Aina and Sofyin strains, and vaccinated with “Tick-E-Vak”. Differences between groups were assessed using the Kruskal–Wallis test; pairwise comparisons were performed using Dunn’s test. The dashed line indicates the lower limit of quantification (LLOQ). Significance levels: * *p* < 0.05, ** *p* < 0.01, *** *p* < 0.001.

In the cerebral hemispheres, upon infection with the Aina strain, significant changes relative to the control were detected for five miRNAs: let-7i *(p* = 0.002), miR-9-5p *(p* = 0.009), miR-30a-5p *(p* < 0.0001), miR-146b-5p *(p* < 0.0001), and miR-181a-5p *(p* = 0.011). Upon infection with the Sofyin strain, a significant difference from the control was detected only for one miRNA, miR-21a-5p *(p* < 0.0001). Upon vaccination with the “Tick-E-Vak” vaccine, a significant difference from the control was likewise detected only for miR-21a-5p *(p* = 0.009) (Figure 5).

Infection with the Aina strain was accompanied by a significant decrease in four miRNAs: let-7i (38.2-fold, *p* = 0.002), miR-9-5p (4.5-fold, *p* = 0.009), miR-30a-5p (6.6-fold, *p* < 0.0001), and miR-146b-5p (12.1-fold, *p* < 0.0001), while miR-181a-5p concentration, in contrast, was significantly increased (3.3-fold, *p* = 0.011). Upon infection with the Sofyin strain and upon vaccination, no significant differences from the control were detected for these five miRNAs. However, the concentrations of let-7i, miR-9-5p, miR-30a-5p, and miR-146b-5p in the Aina-infected group were significantly lower than in the vaccinated group (*p* < 0.0001 for all four comparisons), further emphasizing the specificity of these changes to infection with the Aina strain.

The increase in miR-21a-5p concentration was common to infection with the Sofyin strain and vaccination (8.0-fold, *p* < 0.0001 and 3.0-fold, *p* = 0.009, respectively). Upon infection with the Aina strain, no significant difference from the control was detected for miR-21a-5p. However, its concentration in the Aina group was significantly lower than in both the vaccinated group (3.0-fold, *p* = 0.013) and the Sofyin group (2.7-fold, *p* = 0.017), indicating an opposite direction of changes upon infection with the Aina strain compared with the other two groups.

**Figure 6:**
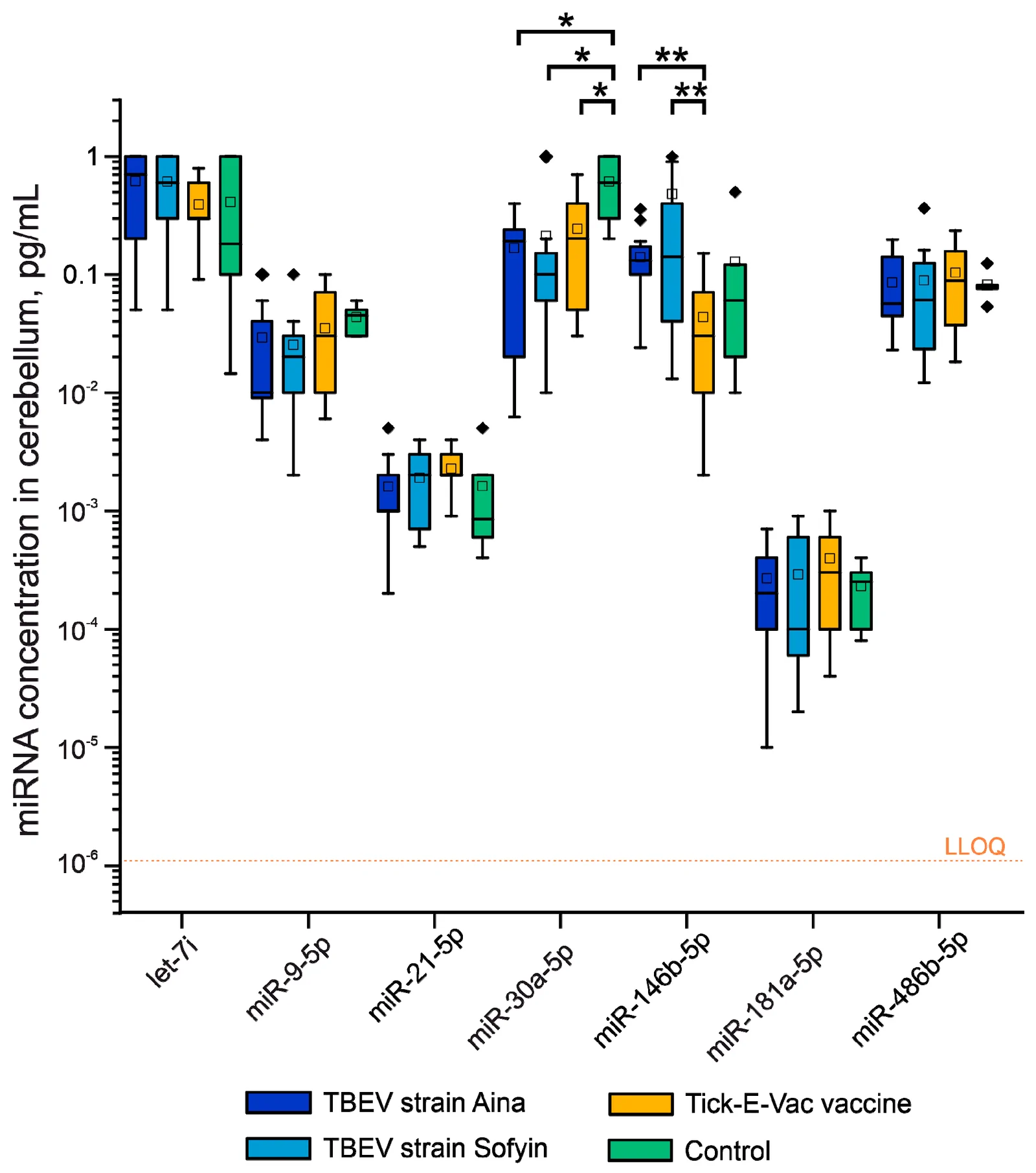
Analysis of miRNA concentrations in the cerebellum of mice infected with the Aina and Sofyin strains, and vaccinated with “Tick-E-Vak”. Differences between groups were assessed using the Kruskal–Wallis test; pairwise comparisons were performed using Dunn’s test. The dashed line indicates the lower limit of quantification (LLOQ). Significance levels: * *p* < 0.05, ** *p* < 0.01.

In the cerebellum, miR-30a-5p concentration was significantly decreased in all three experimental groups: upon infection with the Aina strain *(p* = 0.017), the Sofyin strain *(p* = 0.015), and upon vaccination *(p* = 0.048) (Figure 6). The reduction was most pronounced upon infection with the Sofyin strain and was comparable in magnitude between the Aina-infected and vaccinated groups.

For miR-146b-5p, no significant differences from the control were detected in any experimental group; however, its concentration in both infected groups was significantly higher than in the vaccinated group: 4.3-fold compared with the Aina infection group (*p* = 0.0035) and 4.7-fold compared with the Sofyin infection group (*p* = 0.0025).

**Figure 7:**
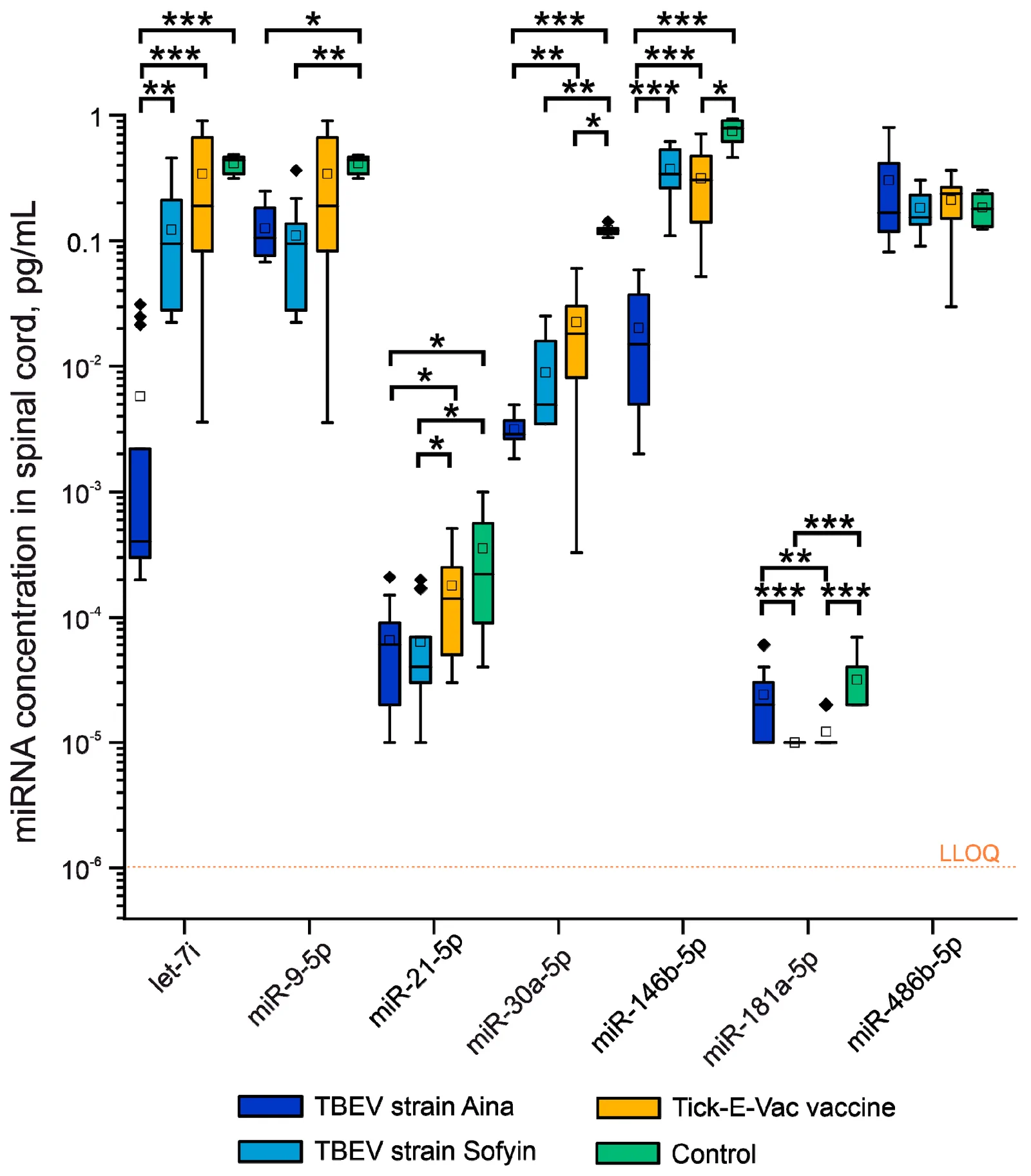
Analysis of miRNA concentrations in the spinal cord of mice infected with the Aina and Sofyin strains, and vaccinated with “Tick-E-Vak”. Differences between groups were assessed using the Kruskal–Wallis test; pairwise comparisons were performed using Dunn’s test. The dashed line indicates the lower limit of quantification (LLOQ). Significance levels: * *p* < 0.05, ** *p* < 0.01, *** *p* < 0.001.

In the spinal cord, miR-30a-5p was altered in all three groups (Figure 7). MiR-9-5p and miR-21a-5p were changed exclusively upon viral infection with both strains. let-7i was reduced only upon infection with the Aina strain. MiR-146b-5p and miR-181a-5p were altered upon vaccination and upon infection with one of the strains: miR-146b-5p with the Aina strain and miR-181a-5p with the Sofyin strain. Notably, all significant changes in the spinal cord represented a decrease in concentration.

MiR-30a-5p was the only miRNA whose decrease was common to both viral infection and vaccination. Its concentration was significantly reduced in all three experimental groups: 42-fold upon infection with the Aina strain (*p* < 0.0001), 24-fold upon infection with the Sofyin strain (*p* = 0.003), and 6.6-fold upon vaccination (*p* = 0.015). The reduction was most pronounced upon infection with the Aina strain and least pronounced upon vaccination.

The decrease in miR-9-5p and miR-21a-5p was specific to viral infection and was observed with both strains. MiR-9-5p was significantly reduced upon infection with the Aina strain (4.2-fold, *p* = 0.011) and the Sofyin strain (4.6-fold, *p* = 0.006), with a comparable magnitude of reduction in both groups. MiR-21a-5p was also significantly reduced upon infection with both strains (Aina: 3.7-fold, *p* = 0.012; Sofyin: 5.5-fold, *p* = 0.011), but the reduction was more pronounced upon infection with the Sofyin strain. Upon vaccination, no significant differences from the control were detected for either miRNA.

The decrease in let-7i was specific to infection with the Aina strain, reaching 1100-fold (*p* < 0.0001). Upon infection with the Sofyin strain, a trend toward decrease was observed (4.6-fold) but did not reach statistical significance (*p* = 0.053), and upon vaccination, no significant differences from the control were detected.

For miR-146b-5p and miR-181a-5p, the decrease was observed upon vaccination and upon infection with one of the strains, but each miRNA responded to a different strain. MiR-146b-5p was significantly reduced upon infection with the Aina strain (53-fold, *p* < 0.0001) and upon vaccination (2.6-fold, *p* = 0.019), whereas upon infection with the Sofyin strain, only a non-significant trend toward decrease was observed (2.3-fold, *p* = 0.057). Conversely, miR-181a-5p was significantly reduced upon infection with the Sofyin strain (2-fold, *p* < 0.0001) and upon vaccination (2-fold, *p* = 0.001), while no significant difference from the control was detected upon infection with the Aina strain.

## 3. Discussion

**Figure 8:**
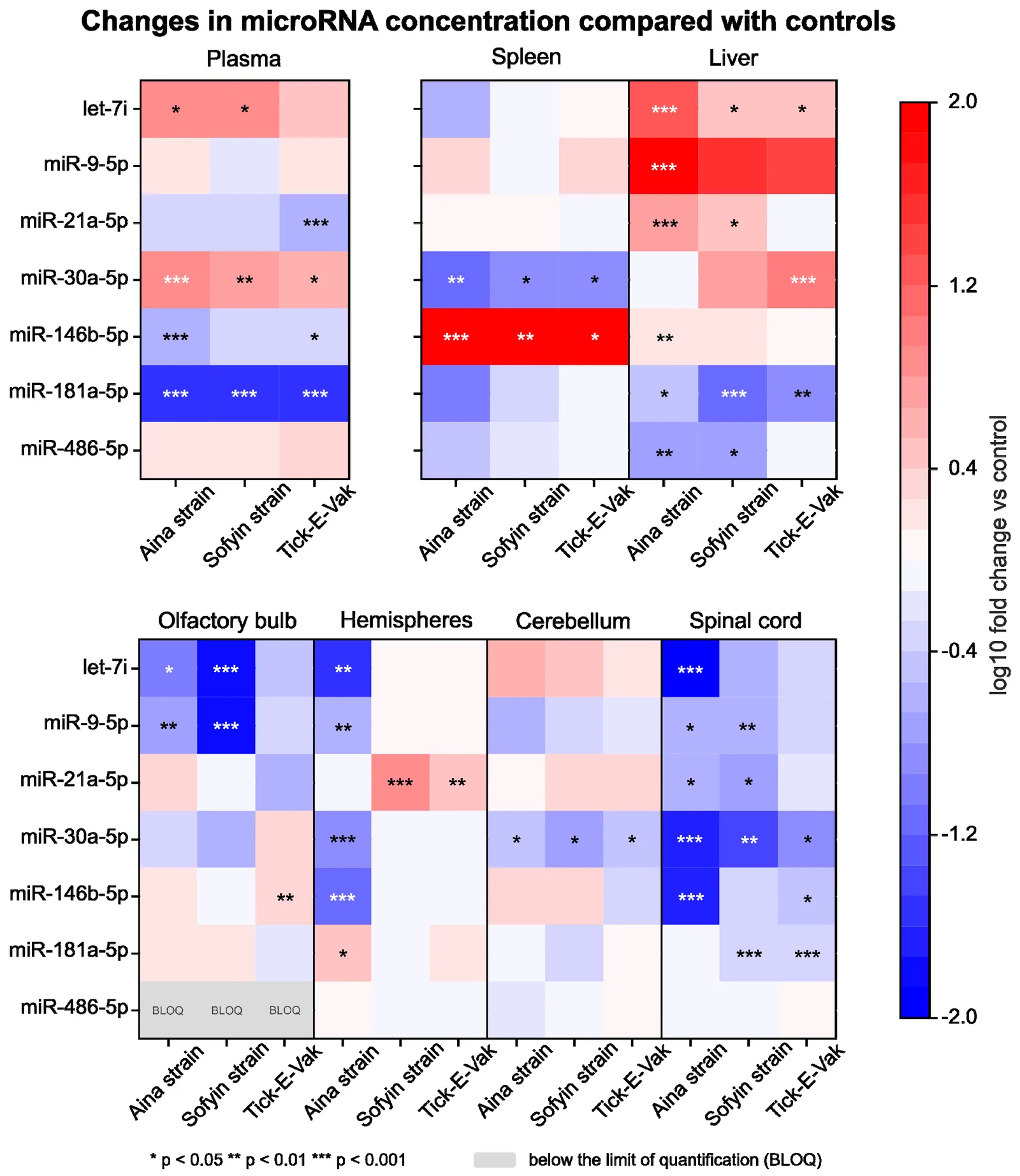
Changes in miRNA concentrations in mouse tissues upon infection with the Aina and Sofyin strains and upon immunization with the “Tick-E-Vak” vaccine compared with the control group. The heatmap displays log-transformed fold changes (log_10_ FC) of median concentrations relative to the control, calculated from log_10_-transformed medians. Red indicates an increase and blue indicates a decrease in concentration. Significance of differences from the control (Dunn’s test with correction): * *p* < 0.05; ** *p* < 0.01; *** *p* < 0.001. Gray cells indicate miRNAs whose concentration was below the lower limit of quantification (LLOQ).

MiRNAs are key post-transcriptional regulators of gene expression that control major signaling pathways of innate and adaptive immunity, ranging from TLR/NF-κB cascade activation to macrophage polarization and autophagy [13,41]. In flavivirus infections, changes in the miRNA profile are involved in regulating the balance between protective antiviral responses and immunopathological damage [12]. This study presents a comparative analysis of changes in the concentrations of seven miRNAs (let-7i, miR-9-5p, miR-21a-5p, miR-30a-5p, miR-146b-5p, miR-181a-5p, miR-486-5p) in blood, peripheral organs (spleen, liver), and CNS structures (olfactory bulb, cerebral hemispheres, cerebellum, spinal cord) of mice upon infection with tick-borne encephalitis virus (Aina and Sofyin strains) and upon immunization with the “Tick-E-Vak” vaccine. All data are summarized in Figure 8. Infection with the Aina strain induced the most pronounced changes in the miRNA profile, substantially exceeding both the effects of immunization and the changes observed upon infection with the Sofyin strain.

### 3.1. Circulating miRNAs: Juxtaposing the Early Stage of Infection in Mice with the Late Stage of Disease in Patients

Plasma miRNAs are extracellular (circulating) and can be considered as biomarkers. Changes in the levels of circulating miRNAs in plasma are determined by the balance between their production and release by cells, on the one hand, and their clearance, on the other. Extracellular miRNAs in plasma enter the bloodstream not only through passive release during cell death, but also through active secretion within exosomes and microvesicles, which is considered a mechanism of intercellular communication [42]. Accordingly, an increase in the plasma concentration of miRNAs may reflect not only the outcome of intracellular regulation of the corresponding pathways, but also an active secretory process by which immune cells transmit regulatory signals to other cells.

The decrease in miR-181a-5p in plasma was pronounced in all three groups. MiR-181a-5p is known as a negative regulator of TLR4/NF-κB signaling [27]. The reduction in miR-181a-5p levels in plasma may reflect decreased production and release of this miRNA by immune cells, in which miR-181a-5p is suppressed to relieve inhibition of the TLR4/NF-κB cascade. Since this process is triggered by both infection and antigenic stimulation by the vaccine, the decrease in miR-181a-5p in plasma may serve as a marker of innate immune activation regardless of viral replication.

In blood plasma, the levels of miR-30a-5p and let-7i were elevated upon infection with both strains, with the most pronounced changes observed for both miRNAs in the Aina strain infection. Both miRNAs are involved in the regulation of innate immune responses, but they target different nodes. MiR-30a-5p suppresses the expression of Beclin-1, a key initiator of autophagosome formation [30,31], whereas let-7i directly targets TLR4 mRNA and suppresses its expression [28,29]. The elevated levels of these miRNAs in plasma may reflect increased production and release by cells in which autophagy is being suppressed and excessive activation of the TLR4/NF-κB cascade is being limited. It should be noted that the increase in miR-30a-5p and let-7i levels in plasma may partially reflect not only enhanced intracellular production, but also active release of these miRNAs within exosomes as mediators of intercellular communication [42,43].

The decrease in miR-21a-5p in blood plasma was vaccine-specific *(p* < 0.001). The p65 subunit of NF-κB directly binds to κB sites in the miR-21 gene promoter and activates transcription of pri-miR-21 [32]. miR-21a-5p suppresses PDCD4 and PTEN, leading to reduced TNF, increased IL-10, and polarization of macrophages toward the M2 phenotype, thereby promoting inflammation resolution [33]. However, miR-21a-5p also suppresses IL12A, attenuating the Th1 response and IFN-γ production [44]. The decrease in miR-21a-5p in plasma upon vaccination may reflect reduced production of this miRNA by cells, which may serve as a mechanism for ensuring an effective Th1 response. During infection, NF-κB-dependent induction of miR-21a-5p, associated with viral replication and tissue damage, may compensate for its suppression, resulting in no significant net change in plasma.

The decrease in miR-146b-5p in plasma was most pronounced upon infection with the Aina strain *(p* < 0.001). MiR-146b-5p is a negative regulator of the TLR/NF-κB cascade [10,11]. The reduction in miR-146b-5p levels in plasma may reflect decreased production and release of this miRNA by immune cells, in which miR-146b is suppressed to relieve inhibition of IRAK1/TRAF6-dependent NF-κB activation. The more pronounced decrease upon infection with the Aina strain may indicate more intensive activation of the proinflammatory cascade during infection with this strain.

We compared our experimental data with miRNA expression profiles in patients [34]. The validity of such cross-species comparison is determined by the degree of evolutionary conservation of the studied miRNAs between mouse (*Mus musculus*) and human (*Homo sapiens*). For all seven miRNAs, the seed regions (nucleotides 2–8) are identical between mouse and human, and five of the seven mature sequences are fully identical (Table S1). Identical miRNA signatures between mouse and human allow effective prediction of targets and assessment of the global impact of individual miRNAs on gene expression [45].

In our previous study [34], we characterized the profile of circulating miRNAs in blood plasma of patients with different clinical forms of TBE (febrile, meningeal, and encephalitic). Among the studied panel, miR-181a-5p and miR-146b-5p are also included in the present study using the mouse model. The changes in the levels of both miRNAs in mice and in patients show opposite directions. In mouse blood plasma, miR-181a-5p and miR-146b-5p were decreased, whereas in TBE patients, both miRNAs were elevated.

A direct comparison of the mouse and human data under matched conditions is impossible in principle. Samples from TBE patients can only be obtained upon hospitalization, i.e., already at the stage of severe clinical symptoms, predominantly within the second (meningoencephalitic) phase of the disease, which is characterized by activation of the adaptive immune arm and immunopathological damage to the CNS [46,47]. The first (viremic/febrile) phase is practically inaccessible for investigation in humans, as patients rarely seek medical attention during this period [46]. In contrast, the mouse model represents an acute monophasic infection, and analysis on day 7 post-infection captures the early stage of active viral replication, viremia, and early neuroinvasion [48,49]. This early stage of the infectious process is unavailable for study in humans. This difference in staging is the key factor explaining the opposite direction of miR-181a-5p and miR-146b-5p changes: in mice, both miRNAs decreased during the phase of maximal NF-κB-dependent proinflammatory activation, when the negative feedback regulators are actively suppressed, whereas in patients they were elevated at the stage of systemic neuroinflammation and immune-mediated CNS damage.

### 3.2. miRNA Responses in Peripheral Organs

The spleen, as the largest secondary lymphoid organ, is a central site of antigen-dependent activation of B and T lymphocytes, and changes in its miRNA profile are expected to reflect the adaptive immune response. The liver plays a central role in the systemic inflammatory response: Kupffer cells, the largest population of tissue macrophages, express Toll-like receptors and secrete TNF-α, IL-6, and IL-1β, while hepatocytes synthesize acute-phase proteins [35]. In the liver and spleen, miRNA changes were substantially more pronounced and organ-specific than in blood. In blood, miRNA levels are determined by the balance of production, release, and clearance [50,51]. For example, in blood plasma, miR-146b-3p was decreased upon infection with the Aina strain, whereas in the spleen, a robust induction of this miRNA was observed in all groups.

In the liver, several miRNAs (let-7i, miR-9-5p, miR-21a-5p, miR-486-5p) were altered exclusively upon infection and remained unchanged upon vaccination. This indicates that these alterations require not merely antigenic stimulation but signals associated with active viral replication. Among these miRNAs, the infection-specific decrease in miR-486-5p is of particular functional interest. This miRNA is a conserved regulator of the PI3K/Akt signaling axis: it represses *PTEN* and *FOXO1*, thereby promoting Akt activity [52,53]. Derepression of these targets in the liver should attenuate PI3K/Akt signaling, which would be consistent with the metabolic reprogramming of hepatic tissue during active viral replication, although this hypothesis requires direct validation of mRNA targets.

For most infection-specific changes, the response to the Aina strain was more pronounced than to the Sofyin strain. The more pronounced response of peripheral organs to Aina infection may reflect differences in pathogenesis between the subtypes. Far Eastern subtype strains closely related to Sofyin have been shown to possess higher neuroinvasiveness with faster crossing of the blood–brain barrier [54]. The Siberian subtype, to which Aina belongs, exhibits a tendency toward persistence, as demonstrated in a mixed infection model in hamsters [55]. These differences may contribute to longer persistence of the Siberian subtype virus in peripheral organs and may explain the more pronounced miRNA changes during Aina infection. An exception is miR-181a in the liver, where the decrease was most pronounced during Sofyin infection, possibly reflecting a stronger activation of innate immunity in hepatocytes during infection with this strain.

A decrease in miR-181a was observed in all studied compartments, including blood, peripheral organs, and all experimental groups, making it the most consistent change in this study. Such universality suggests that suppression of miR-181a, a negative regulator of TLR4/NF-κB [27], may represent a common, tissue-independent mechanism of innate immune response activation triggered by both infection and antigenic stimulation by the vaccine.

### 3.3. Central Nervous System: Region-Specific miRNA Regulation

The olfactory bulbs are considered one of the primary gateways of neuroinvasion. The virus enters the CNS through the olfactory neuroepithelium and spreads along the olfactory tract [36,37]. The cerebral hemispheres are the main zone of neuronal damage, as TBEV infects pyramidal neurons of the cortex and hippocampus [38]. The cerebellum is involved in the development of rhombencephalitis [38]. The spinal cord is affected in myelitis, which is one of the clinical manifestations of severe TBEV [39,40].

In the CNS, unlike peripheral organs, several miRNAs (let-7i, miR-9-5p, miR-21a-5p, miR-146b-5p) changed exclusively during infection and remained unchanged after vaccination in the olfactory bulb and spinal cord. This is consistent with the induction of neuroinflammatory changes in the CNS by active viral replication and associated tissue damage, although viral load was not directly measured in this study. However, vaccination did cause some CNS-specific changes: an increase in miR-146b-3p in the olfactory bulb and miR-21a-5p in the hemispheres. This may reflect activation of immune surveillance in the CNS in response to systemic antigenic stimulation.

The ratio of responses to the Aina and Sofyin strains in the CNS was fundamentally different from that in peripheral organs. In the spleen and liver, changes during Aina infection were more pronounced than during Sofyin infection. In the CNS, this ratio depended on the brain region examined: in the olfactory bulb, which serves as a gateway for neuroinvasion, more pronounced changes were caused by Sofyin (decrease in let-7i and miR-9-5p), whereas in the hemispheres and spinal cord, Aina dominated. This distribution may reflect different routes and kinetics of neuroinvasion. The Sofyin strain, which possesses higher neuroinvasiveness [54,56], causes more pronounced changes in the olfactory bulb, which is particularly vulnerable to neurotropic flaviviruses [38,57]. In contrast, more profound changes in the spinal cord during Aina infection may indicate spread of the virus along the neuroaxis, consistent with data on the progression of neuropathology in severe TBE infection [58].

A decrease in miR-30a was observed in all CNS structures, and in the cerebellum it was significant in all three groups. This may reflect activation of autophagy in neurons and/or microglia regardless of the type of stimulus [30,31]. In the spinal cord, the magnitude of miR-30a decrease declined in the following order: Aina > Sofyin > vaccine. A similar direction of miR-30a decrease in the spleen and CNS, but the opposite in blood, supports the tissue-specific nature of autophagy regulation.

Let-7i and miR-9-5p showed coordinated infection-specific changes in the CNS. Both miRNAs decreased during infection in the olfactory bulb, hemispheres, and spinal cord, with the greatest changes affecting the structures most vulnerable to neuroinvasion. The coordinated decrease in let-7i and miR-9-5p may thus reflect a common mechanism of innate immune response modulation in the CNS during flaviviral infection.

### 3.4. Biomarker Potential of the Identified miRNAs

Circulating plasma miRNAs represent an attractive class of candidate non-invasive biomarkers for infectious diseases, as they are stable in biological fluids and their profile changes in response to infection [59,60]. The present study in a mouse model allows identification of several groups of miRNAs of potential interest for clinical application in humans, pending subsequent validation.

miR-181a-5p and miR-30a-5p, which changed in the same direction across all three experimental groups, may be considered as markers of innate immunity activation. The decrease in miR-181a-5p was the most reproducible change in this study, observed in all compartments. Notably, the direction of miR-181a-5p change in mice (decrease during the acute phase) and in patients (increase during the second phase) was opposite, reflecting phase dependence of the plasma profile and highlighting the need to account for the disease stage in clinical interpretation.

An increase in let-7i was observed only during viral infection and was absent after vaccination, making it a potential marker for distinguishing active infection. Notably, the designation of let-7i and miR-9-5p as candidate markers associated with TBEV infection or CNS involvement is at this stage preliminary: viral load, tissue virus distribution, and histopathological changes were not assessed in this study.

miRNAs whose plasma changes showed a gradient of magnitude between strains are also promising as markers of infection severity. Since the miRNA response to the Aina strain suggests the most intense activation of the proinflammatory cascade, the gradient of miR-30a-5p and miR-146b-5p changes may reflect the degree of immune response severity.

However, when assessing biomarker potential, a fundamental limitation must be considered: plasma miRNAs do not reflect the complete picture of changes occurring in the body. As the present data show, the direction of changes in plasma often does not match that in tissues. For example, miR-146b-5p decreased in plasma during Aina infection, but a massive induction was observed in the spleen; miR-30a-5p increased in plasma but decreased in all CNS structures. This means that the plasma miRNA profile is an integral but incomplete reflection of tissue processes, and clinical interpretation of changes in an individual miRNA in blood without considering the tissue context may be misleading.

### 3.5. Advantages and Limitations of the Study

Data obtained in the mouse model provide several unique opportunities unavailable in clinical studies. First, the mouse model allows investigation of early miRNA profile changes during the acute phase of infection, before the development of clinically manifest neurological disease, which is particularly valuable for studying neurotropic infections where access to early disease stages in humans is extremely limited [61]. This is critically important, as early diagnosis and understanding of initial pathogenetic mechanisms determine the possibilities for therapeutic intervention. Second, the experimental model ensures strictly controlled conditions. Third, the mouse model allows direct comparison of two strains under identical conditions, enabling identification of strain-specific features of miRNA regulation determined by genetic differences between viruses rather than host factors. Fourth, the model provides access to tissues (brain, spleen, lymph nodes) that are unavailable from living patients, allowing correlation of miRNA expression changes with viral load, histopathological changes, and viral distribution in organs.

The present study also has several limitations. First, miRNA concentrations were measured in tissue homogenates and blood plasma, which does not allow determination of the cellular source of the changes. Extracellular miRNAs circulate predominantly within extracellular vesicles and in complexes with proteins, and individual tissues contribute unequally to the circulating miRNA pool. Therefore, changes detected in tissue homogenates cannot be directly extrapolated to the release of these miRNAs into the bloodstream. Furthermore, the directionality of miRNA exchange between blood and brain could not be established. Second, the study was conducted at a single time point, which does not allow assessment of the dynamics of miRNA changes during the development of the immune response. Third, miRNA concentrations were the only molecular readout in this study, and the relevant signaling pathways and their molecular mediators were not analyzed. Therefore, the pathway-level interpretations presented in the Discussion remain hypothetical and require experimental validation using direct measurements of pathway activity. Fourth, viral load, viral distribution in tissues, and histopathological changes were not assessed, which does not allow direct correlation of miRNA changes with viral replication and neurovirulence.

## 4. Materials and Methods

### 4.1. Blood and Organ Collection in Mice

The study protocol was reviewed and approved by the Inter-Institutional Bioethics Committee at the Institute of Cytology and Genetics, Siberian Branch of the Russian Academy of Sciences (protocol No. 186, 4 September 2024). All experiments were conducted in strict accordance with the American Veterinary Medical Association Guidelines for the Euthanasia of Animals (AVMA, 2020) and the ARRIVE Guidelines [62]. Throughout the experiment, the rules for the use and housing of laboratory animals were followed to minimize animal suffering and ensure the reliability of experimental data.

BALB/c mice were used in this study. Animals were housed with free access to food and water, at a temperature of 22 ± 1 °C, relative humidity of 60%, and a 12-h light–dark cycle, with 2–4 animals per cage.

The experiment included 4 groups of mice, 8 animals per group:

- Sofyin-Chumakov infection group (Far Eastern TBEV subtype);
- Aina infection group (Siberian TBEV subtype);
- Vaccinated group—immunization with the “Tick-E-Vak” vaccine;
- Control group—mice immunized with PBS.

Mice in the infection groups received the corresponding tick-borne encephalitis virus strain (Sofyin-Chumakov or Aina) subcutaneously in a volume of 100 μL at a dose of 10^4^ plaque-forming units (PFU). Mice in the vaccinated group received the “Tick-E-Vak” vaccine (Chumakov Federal Scientific Center for Research and Development of Immune-and-Biological Products, FMBA of Russia) intramuscularly into the thigh muscle at a dose of 100 μL per mouse (1:10 dilution of the human therapeutic dose) as a single administration. Animals in the control group received sterile saline intramuscularly at 100 μL per mouse. Tissues were collected 7 days after infection or vaccine administration.

Biological samples were collected by decapitation. Blood was collected into tubes containing 4% sodium citrate at a volume ratio of 1:9 and centrifuged at 3000× *g* for 10 min. Plasma was stored at −20 °C. CNS tissues (cerebral hemispheres, olfactory bulbs, cerebellum, spinal cord), as well as liver and spleen, were dissected and stored at −20 °C.

### 4.2. MiRNA Extraction and Stem-Loop RT-qPCR

miRNA extraction was performed using reagents from the “Total RNA and microRNA Isolation Kit” (LRU-100-50, Biolabmix, Novosibirsk, Russia) according to the manufacturer’s protocol. Quantification of miRNAs by reverse transcription using stem-loop primers (Stem-Loop RT-qPCR) was performed as described [34,63]. The sequences of the primers used are listed in Table 1. MiRNA quantification in blood plasma was performed by absolute quantitative analysis. For each miRNA, a calibration curve was constructed using tenfold serial dilutions of the corresponding synthetic oligonucleotide (with a sequence identical to the mature miRNA) in the concentration range from 10^−2^ to 10^−9^ ng/μL. Tissue miRNA concentration was normalized to 100 μg of sample. A representative standard curve (miR-146b-5p) is shown in Figure S1; the standard curves for all seven miRNAs were similar. Standard curves were generated using serial dilutions of synthetic miRNA oligonucleotides (ng/μL range); the derived concentrations are expressed in pg/mL for clarity of axis scaling.

### 4.3. Statistical Analysis

Most variables had a non-normal distribution (Shapiro–Wilk test); therefore, nonparametric tests were used to analyze the significance of differences. The significance of differences between multiple groups was calculated using the Kruskal–Wallis test. For pairwise comparisons, the Mann–Whitney U test was used. Post hoc pairwise comparisons of all group pairs were performed using Dunn’s test. To control false discoveries in multiple comparisons, the Benjamini–Hochberg correction was applied, controlling the expected false discovery rate (FDR). Differences were considered statistically significant at padj < 0.05. Calculations were performed in Python 3 using the SciPy library (Kruskal–Wallis test), scikit-posthocs (Dunn’s test), and statsmodels (Benjamini–Hochberg correction). Statistically significant pairwise differences (padj < 0.05) are indicated by asterisks: * *p* < 0.05; ** *p* < 0.01; *** *p* < 0.001.

miRNA concentrations in blood plasma were calculated in pg/mL. Samples with a concentration below the limit of quantification (LOQ = 1 × 10^−6^ pg/mL) were designated as BLOQ (below limit of quantification). For statistical analysis, these samples were assigned a value equal to half the limit of detection (LOD/2 = 5 × 10^−7^ pg/mL).

Fold changes in miRNA concentrations were calculated as the ratio of log10-transformed median values in the experimental group and the control group, with back-transformation to the linear scale: FC = 10^(median_log10_group − median_log10_control). For decreases, the reciprocal of FC was used.

## 5. Conclusions

The present study presents a systematic comparative analysis of tissue-specific miRNA profile changes during infection with two tick-borne encephalitis virus strains representing epidemiologically significant subtypes (Sofyin and Aina) and after vaccination with the “Tick-E-Vak” vaccine. Assessment of the concentration of seven immunoregulatory miRNAs in seven compartments (blood plasma, peripheral organs, and four CNS structures) revealed a number of patterns relevant to understanding the pathogenesis of TBEV infection and the development of biomarker approaches.

miR-181a-5p is a negative regulator of TLR4/NF-κB signaling. Its decrease was the most reproducible change in this study, observed in all tissues and across all three experimental groups. This universality suggests that derepression of the proinflammatory cascade through suppression of miR-181a-5p may represent a common mechanism of immune activation, independent of both the type of stimulus (infection or vaccination) and the tissue context, although this interpretation requires direct validation of pathway activity. Two other miRNAs (miR-30a-5p and miR-146b-5p) changed in all studied tissues. However, the direction of their changes differed substantially between compartments: in plasma, miR-30a-5p increased, whereas in the CNS it decreased; miR-146b-5p decreased in plasma but increased in the spleen. This opposite directionality may reflect redistribution of miRNAs between the tissue and circulating pools during immune activation and is important for the interpretation of plasma biomarkers: the profile of circulating miRNAs is not a simple projection of tissue changes.

In peripheral organs and the CNS, several miRNAs changed in opposite directions. Most notably, let-7i and miR-9-5p decreased coordinately in the CNS structures most vulnerable to neuroinvasion (olfactory bulb, spinal cord), whereas in the liver the same miRNAs significantly increased. This suggests the existence of different mechanisms of immune response regulation in peripheral organs and the CNS and is consistent with the neuroinvasive nature of TBEV. Infection-specific changes in let-7i and miR-9-5p in the CNS, which were absent after vaccination, make them candidate markers associated with TBEV infection and CNS involvement. However, in the absence of direct measurements of viral load and tissue pathology, these changes should be interpreted as infection-associated rather than replication-specific.

Infection with the Aina strain (Siberian subtype) caused more pronounced miRNA changes in both peripheral organs and the CNS than infection with the Sofyin strain (Far Eastern subtype). The more pronounced changes in miRNA levels during Aina infection are consistent with data showing that Siberian TBEV isolates demonstrate virulence in the mouse model comparable to or exceeding that of Far Eastern strains [64]. Differences in the miRNA response between strains may reflect strain-specific features of replication and tropism, similar to those described for other pairs of TBEV strains with different pathogenicity [48]. At the same time, molecular genetic studies show that Sofyin-like strains possess genetically determined advantages in neuroinvasiveness [54]. However, direct comparisons of neuroinvasiveness between the Far Eastern and Siberian subtypes in the literature are limited.

Comparison of the miRNA response to natural infection and vaccination revealed both common and specific components. Common changes (decrease in miR-181a-5p, changes in miR-30a-5p and miR-146b-5p) are consistent with activation of basic innate immunity signaling pathways induced by both antigenic stimulation and active viral replication. Infection-specific changes (increase in let-7i and miR-21a-5p in the liver, decrease in let-7i and miR-9-5p in the CNS) appear to require signals associated with active viral replication and are absent after vaccination. Vaccination-specific decrease in miR-21a-5p in plasma and increase in miR-30a-5p in the liver suggest qualitative differences between the immune response induced by the inactivated vaccine and the response to natural infection. These differences are consistent with existing data showing that immunity induced by inactivated vaccines differs from immunity following natural infection in the spectrum of engaged effector mechanisms.

The obtained results support the potential of using miRNAs as non-invasive biomarkers of tick-borne encephalitis. MiR-181a-5p and miR-30a-5p, which changed in the same direction across all groups, may be considered as markers of innate immunity activation. The increase in let-7i in plasma, observed only during viral infection and absent after vaccination, may serve as a candidate marker for distinguishing active infection from post-vaccinal immunity. The gradient of miR-30a-5p and miR-146b-5p changes in plasma between strains may reflect the severity of infection. Comparison of the experimental data with the previously characterized profile of circulating miRNAs in patients with different clinical forms of tick-borne encephalitis supports the translational relevance of the identified markers and supports the feasibility of further validation in clinical cohorts.

Thus, the results of the present study demonstrate that TBEV infection and vaccination induce tissue-specific, strain-specific, and qualitatively distinct changes in the miRNA profile, reflecting different aspects of the immune response. The presented map of tissue-specific miRNA responses expands the understanding of TBEV infection pathogenesis, identifies candidates for non-invasive biomarkers for differential diagnosis of infection and vaccination, assessment of severity, and evaluation of CNS involvement, and supports the feasibility of further studies on miRNA dynamics during the development of the immune response, as well as their validation in clinical cohorts of patients with tick-borne encephalitis.

## Figures and Tables

**Table 1:** List of primers and probes used in this study.

miRNA	SL-Primer	Forward Primer
miR-9-5p	5′–GTTGGCTCTGGTGCAGGGTCCGAGGTATTCGCACCAGAGCCAACTCATAC–3′	5′–GTTGGTCTTTGGTTATCTAGCT–3′
miR-30a-5p	5′–GTTGGCTCTGGTGCAGGGTCCGAGGTATTCGCACCAGAGCCAACCTTCCA–3′	5′–GGGTGTAAACATCCTCGAC–3′
miR-181a-5p	5′–GTTGGCTCTGGTGCAGGGTCCGAGGTATTCGCACCAGAGCCAACACTCAC–3′	5′–GGAACATTCAACGCTGTCG–3′
miR-146b-5p	5′–GTTGGCTCTGGTGCGGGTCCGAGGTATTGCACCAAGAGCCAACCAGCCT–3′	5′–GTTTTTCGTGAGAACTGAATTCCAT–3′
miR-486-5p	5′–GTTGGCTCTGGTGCAGGGTCCGAGGTATTCGCACCAGAGCCAACCTCGGG–3′	5′–GTTTCCTGTACTGAGCTGC–3′
miR-21a-5p	5′–GTTGGCTCTGGTGCAGGGTCCGAGGTATTCgCACCAGAGCCAACAGTCAA–3′	5′–GTTGTAGCTTATCAGACTGATG–3′
let-7i	5′–GTTGGCTCTGGTGCAGGGTCCGAGGTATTCGCACCAGAGCCAACAACAGC–3′	5′–GTTTGGTGAGGTAGTAGTTTGT–3′

## Data Availability

Data is contained within the article.

## Supplementary Materials

The following supporting information can be downloaded at https://www.mdpi.com/article/10.3390/ncrna12050042/s1, Table S1: Comparison of mature miRNA sequences of a mouse panel with human orthologs. Figure S1. Representative standard curve for miR-146b-5p determined by stem-loop RT-qPCR using serial tenfold dilutions of synthetic oligonucleotide standards (10^−2^–10^−7^ ng/µL, double replicate per point).
