# Supplementary material for: Tissue-Specific miRNA Responses to Tick-Borne Encephalitis Virus Strains in Mice
Source: Noncoding RNA. 2026 Oct 20;12(5):42. doi: 10.3390/ncrna12050042 (PMC13708369; doi:10.3390/ncrna12050042)
Supplement: Supplementary file 1 [file ncrna-12-00042-s001.zip › ncrna-4579970-supplementary.pdf]

Table S1. Comparison of mature miRNA sequences of a mouse panel with human orthologs.

miRNA	Sequence, mouse (5'→3')**	Sequence, human (5'→3')	Seed (2–8)	Identity*	Note
miR-9-5p	TCTTTGGTTATC TAGCTGTATGA	TCTTTGGTTATC TAGCTGTATGA	CTTTGGT	100%	Identically
miR-30a-5p	TGTAAACATCCT CGACTGGAAG	TGTAAACATCCT CGACTGGAAG	GTAAACA	100%	identically
miR-181a-5p	AACATTCAACG CTGTCGGTGAGT	AACATTCAACG CTGTCGGTGAGT	ACATTCA	100%	identically
miR-146b-5p	TGAGAACTGAA TTCCATAGGCT	TGAGAACTGAA TTCCATAGGCTG	GAGAACT	100%	humans have +G at the 3' end
miR-486a-5p	TCCTGTACTGAG CTGCCCCGAG	TCCTGTACTGAG CTGCCCCGAG	CCTGTAC	100%	the mouse has two paralogs (miR-486a, miR-486b), both identical to humans.
miR-21a-5p	TAGCTTATCAG ACTGATGTTGA	TAGCTTATCAG ACTGATGTTGAC T	AGCTTAT	100%	humans have +CT at the 3' end
let-7i	TGAGGTAGTAG TTTGTGCTGTT	TGAGGTAGTAG TTTGTGCTGTT	GAGGTAG	100%	identically

* Identity is calculated based on the length of the shorter (mouse) sequence; differences are localized exclusively at the 3' end and do not affect the seed region.

**Data source: miRBase release 22.1 (http://www.mirbase.org/).

Comparison of mature sequences showed complete identity of the seed region (nucleotides 2–8, which determine the specificity of binding to mRNA targets) between mouse and human variants for all seven miRNAs (table in supplementary materials). Five of the seven mature sequences are fully identical between the two species. Two miRNAs (miR-146b-5p and miR-21a-5p) differ only by additional nucleotides at the 3' end, which does not affect the seed region and, consequently, does not influence the spectrum of recognized targets. It should be noted that miR-486 in the mouse is represented by two paralogs (distinct genomic loci that are processed into the same mature miRNA, mmu-miR-486a-5p and mmu-miR-486b-5p). The sequence conservation of miRNAs is accompanied by conservation of their targets and functions

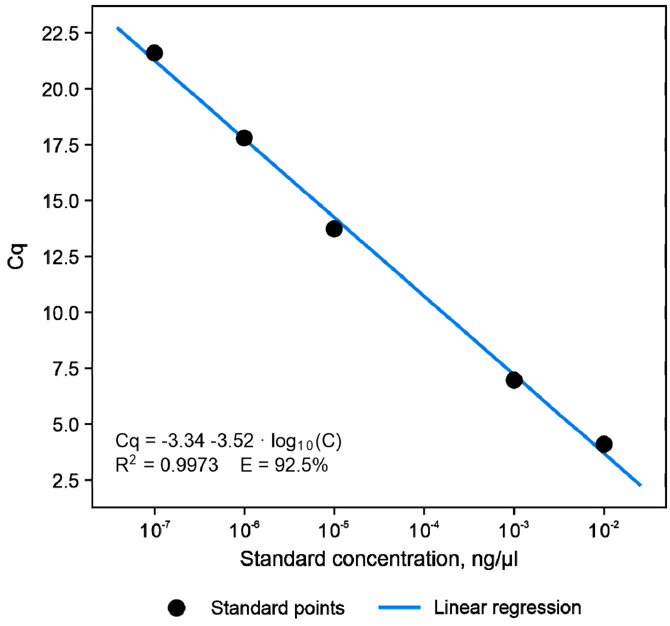

Figure S1. Representative standard curve for miR-146b-5p determined by stem-loop RT-qPCR using serial tenfold dilutions of synthetic oligonucleotide standards (10^{-2}–10^{-7} ng/µl, double replicate per point).
